# Supplementary material for: Asynchronous magnetic resonance elastography: Shear wave speed reconstruction using noise correlation of incoherent waves
Source: Magn Reson Med. 2022 Oct 27;89(3):990–1001. doi: 10.1002/mrm.29502 (PMC9792433; doi:10.1002/mrm.29502)
Supplement: Supplementary file 1 — DATA S1 MATLAB code used to generate the numerical simulations of Figure 1 [file MRM-89-990-s002.zip › k-Wave/helpfiles/example_cpp_io_in_parts.html]

Saving Input Files In Parts Example (k-Wave)


# Saving Input Files In Parts Example

This example demonstrates how to save the HDF5 input files required by the C++ code in parts. It builds on the Running C++ Simulations Example.

- Open the file in the MATLAB Editor
- Run the file in MATLAB

## Contents

- Saving the medium, source, and sensor
- Saving the grid and file attributes

## Saving the medium, source, and sensor

When performing simulations with very large grid sizes, out-of-memory errors can sometimes be encountered when using `kspaceFirstOrder3D` to generate the input files for the C++ code. This is because all input matrices are first created in memory before saving them to disk. It is also possible to create the input HDF5 files in parts, where each input matrix is generated, saved to disk, and then cleared from memory sequentially, rather than all at once. Variables are written to the input file by first casting them to the correct data type, and then using the `writeMatrix` function. The required data types are stored as the literals `MATRIX_DATA_TYPE_MATLAB` and `INTEGER_DATA_TYPE_MATLAB` defined in `private/getH5Literals.m`. These can be loaded to the workspace using

```
% load HDF5 constants
run([getkWavePath 'private/getH5Literals']);
```

The variables stored in the HDF5 file must be given the correct names and must have the correct data type and matrix structure. A comprehensive list of the required input variables and their format is given in the k-Wave Manual. For example, the syntax for casting and saving the sound speed is:

```
% make sure the input is in the correct data format
eval(['c0 = ' MATRIX_DATA_TYPE_MATLAB '(c0);']);
    
% save the sound speed matrix
writeMatrix([pathname input_filename], c0, 'c0');
```

Note, the interpolation functions in MATLAB used to calculate the values of the density on a staggered grid, become very inefficient for large matrices. In this case, it is better to use alternative methods to calculate the density values on the staggered grids.

When using a time-varying pressure or velocity source, the source mask must be defined as a 1D list of linear grid indices that correspond to the grid points that will act as source points. For example, if the source is square piston, the source mask can be created and written to the input file as follows:

```
% define a square source mask facing in the x-direction using the
% normal k-Wave syntax
p_mask = false(Nx, Ny, Nz);
p_mask(1 + pml_x_size, Ny/2 - source_y_size/2:Ny/2 + source_y_size/2, Nz/2 - source_z_size/2:Nz/2 + source_z_size/2) = 1;

% find linear source indices
p_source_index = find(p_mask == 1);
p_source_index = reshape(p_source_index, [], 1);

% make sure the input is in the correct data format
eval(['p_source_index = ' INTEGER_DATA_TYPE_MATLAB '(p_source_index);']);

% save the source index matrix
writeMatrix([pathname input_filename], p_source_index, 'p_source_index');
```

The corresponding time-varying pressure source is created in the normal fashion and then written to the `p_source_input` within the input file. Note, when using `writeMatrix` instead of `kspaceFirstOrder3D` to create the input file, the source scaling must be manually applied.

```
% define a time varying sinusoidal source
p_source_input  = source_strength .* sin(2 * pi * source_freq * (0:(Nt - 1)) * dt);

% apply an cosine ramp to the beginning to avoid startup transients
ramp_length = round((2*pi/source_freq)/dt);
p_source_input(1:ramp_length) = p_source_input(1:ramp_length).*(-cos( (0:(ramp_length-1))*pi/ramp_length ) + 1)/2;

% scale the source magnitude to be in the correct units for the code
p_source_input = p_source_input .* (2 * dt ./ (3 * c_source * dx));

% cast matrix to single precision
eval(['p_source_input = ' MATRIX_DATA_TYPE_MATLAB '(p_source_input);']);

% save the input signal
writeMatrix([pathname input_filename], p_source_input, 'p_source_input');
```

The sensor mask must be defined as a 1D list of linear grid indices in the same way as the source masks:

```
% define a sensor mask through the central plane
sensor_mask = false(Nx, Ny, Nz);
sensor_mask(:, :, Nz/2) = 1;

% extract the indices of the active sensor mask elements
sensor_mask_index = find(sensor_mask);
sensor_mask_index = reshape(sensor_mask_index, [], 1);

% make sure the input is in the correct data format
eval(['sensor_mask_index = ' INTEGER_DATA_TYPE_MATLAB '(sensor_mask_index);']);

% save the sensor mask
writeMatrix([pathname input_filename], sensor_mask_index, 'sensor_mask_index');
```

Alternatively, the sensor mask can be defined as a list of cuboid corners as described in the k-Wave manual.

## Saving the grid and file attributes

After the medium, source, and sensor properties have been written to the input file, a number of additional grid variables, input flags, and file attributes must also be added. This can be done by using the functions `writeGrid`, `writeFlags`, and `writeAttributes`.

```
% write grid parameters
writeGrid([pathname input_filename], [Nx, Ny, Nz], [dx, dy, dz], ...
    [pml_x_size, pml_y_size, pml_z_size], [pml_x_alpha, pml_y_alpha, pml_z_alpha], ...
    Nt, dt, c_ref);

% write flags
writeFlags([pathname input_filename]);

% set additional file attributes
writeAttributes([pathname input_filename]);
```

After the input file is generated, the C++ code can be called in the same way as the Using the C++ Code Example.
